# Perirhinal Cortex LTP Does Not Require Astrocyte BDNF-TrkB Signaling

**DOI:** 10.3390/cells11091501

**Published:** 2022-04-29

**Authors:** Beatrice Vignoli, Marco Canossa

**Affiliations:** 1 Department of Physics, University of Trento, 38123 Povo (TN), Italy; 2 Department of Cellular, Computational and Integrative Biology (CIBIO), University of Trento, 38123 Povo (TN), Italy; marco.canossa@unitn.it

**Keywords:** astrocyte microdomains, synaptic strengthening, neuron–astrocyte communication

## Abstract

Neurons release and respond to brain-derived neurotrophic factor (BDNF) with bursts of brain activity. BDNF action is known to extend to peri-synaptic astrocytes, contributing to synaptic strengthening. This implies that astrocytes have a set of dynamic responses, some of which might be secondary to activation of the tropomyosin tyrosine kinase B (TrkB) receptor. Here, we assessed the contribution of BDNF to long-term synaptic potentiation (LTP), by specifically deleting TrkB in cortical astrocytes. TrkB deletion had no effect on LTP induction, stabilization and maintenance, indicating that TrkB signaling in astrocytes is extraneous to transducing BDNF activity for synaptic strengthening.

## 1. Introduction

An essential property of the mammalian brain is the skill to adapt its function based on experience, and thus, to adjust resulting behavior. By prolonging the strength of synaptic transmission, momentary experiences can be integrated into the neuronal circuits as persistent memory traces. Thus, synaptic strengthening is a central mechanism that wires memory functions [1].

Astrocytes have been found to be key players in synaptic plasticity [2,3,4,5,6]. They express receptors that sense the release of neurotransmitters/neuromodulators from neighboring active synapses and provide intracellular signaling to modulate synaptic strengthening and behavioral responses [7]. Contextually, astrocytes express receptors for brain-derived neurotrophic factor (BDNF); however, their participation in the regulation of synaptic long-term potentiation (LTP) is a hotly debated topic [8,9]. We previously demonstrated that in layers II/III in the perirhinal cortex, astrocytes recruited the pan-neurotrophin receptor p75 (p75^NTR^) for LTP maintenance and memory consolidation [10,11]. Here, we extend our investigation to assess whether the BDNF receptor tropomyosin tyrosine kinase B (TrkB) is involved in this process. Once released from neurons, BDNF has the potential to trigger TrkB signaling on peri-synaptic astrocytes and transduce responses, collectively regulating LTP. We addressed this hypothesis in perirhinal cortex astrocytes involved in LTP maintenance.

The major challenge when studying the effects of astrocytes on synaptic strengthening is isolating the glia-specific signaling components that contribute to that of neurons in the tripartite synapse. Using various strategies, including pharmacological and genetic manipulation and gene expression using viral vectors, we depleted TrkB expression from astrocytes and addressed the contribution of its downstream signaling to cortical LTP. The absence of astrocyte TrkB resulted in no alteration in cortical LTP, indicating that TrkB signaling in astrocytes is unrelated to synaptic strengthening.

## 2. Materials and Methods

### 2.1. Animals

TrkB^lox/lox^-Cre mice were generated by crossing TrkB^lox/lox^ mice, kindly provided by Rudiger Klein (Max Planck Institute of Neurobiology, Munich, Germany), with GLAST-CreER^T2^-ROSA26 mice, kindly provided by Prof. M. Gotz (LMU, Munich, Germany). Animals were housed in a 12 h light/dark cycle with unrestricted access to food and water. Adult male TrkB^lox/lox^-Cre mice and control littermates were treated with 1 mg of tamoxifen dissolved in corn oil twice a day for 5 consecutive days.

### 2.2. Stereotaxic Surgery

For virus delivery, on the last day of tamoxifen administration, the mice were deeply anesthetized, and a viral particle (1.5 µL in volume) was infused into the perirhinal cortex from each hemisphere (coordinates from Bregma: anteroposterior—2 mm, lateral ±4.2 mm, ventral +2.8 mm). Viral delivery was obtained through the insertion of capillary glasses (WPI) connected to a manual syringe pump (Narishige, Tokyo, Japan). Mice were allowed to recover and were housed in standard cages until the day of sacrifice.

### 2.3. Viral Vectors

LV-GFP^stop^: for Cre-dependent expression, lentiviral vectors were produced starting from pLV, which drives gene expression under the cytomegalovirus (CMV) promoter. A gene-cassette including an eGFP encoding sequence was flanked by two modified loxP sites (lox2272). This prevented the formation of unwanted ATG start codons after Cre-mediated recombination. The eGFP expression was stopped by two stop codons. Next, a single EcoRV-blunt cloning site, an IRES2 (internal ribosomal entry site 2) followed by TandemTomato (tdTomato), was cloned in the 5′ direction, before the WPRE lentiviral vector element. This construct shows strong GFP expression and weak expression of IRES2-tdTomato. Under Cre-mediated recombination, the eGFP was lost and the IRES2-tdTomato cassette was under control of the promoter. pAAV.GFAP.Cre.WPRE.hGH (AAV-GFAP-Cre) was a gift from James M. Wilson (Addgene viral prep # 105550-AAV5; http://n2t.net/addgene:105550; RRID:Addgene_105550); pZac2.1 gfaABC1D-tdTomato (AAV-GFAP-tdTomato) was a gift from Baljit Khakh (Addgene viral prep # 44332-AAV5; http://n2t.net/addgene:44332; RRID:Addgene_44332).

### 2.4. Slice Preparation

Slices from the perirhinal cortex were prepared from TrkB^lox/lox^-Cre mice. Control littermates were injected with LV-GFP^stop^ and TrkB^lox/lox^ mice were co-injected with AAV-GFAP-Cre and AAV-GFAP-tdTomato. The brains were removed and placed in cold, oxygenated (95% O_2_ and 5% CO_2_), artificial cerebrospinal fluid (ACSF), containing 124.0 mM NaCl, 4.4 mM KCl, 1 mM NaH_2_PO_4_, 2.5 mM CaCl_2_, 1.3 mM MgCl_2_, 26.2 mM NaHCO_3_, 10 mM glucose and 2 mM L-ascorbic acid. Horizontal cortex slices (300 μm-thick) were prepared using a vibratome and were maintained in a chamber containing oxygenated ACSF at room temperature. After a minimum recovery period of 1 h, a single slice was transferred into a submersion recording chamber.

### 2.5. Electrophysiological Recording

After the recovery slices were perfused (3 mL/min) with oxygenated ACSF at 32 ± 0.2 °C, square current pulses (duration 0.2 ms) were applied every 30 s (0.033 Hz) using a stimulus generator (WPI, stimulus isolator A360) connected through a stimulus isolation unit to a concentric bipolar electrode (40–80 KU, FHC) positioned in layers II/III on the temporal side of the rhinal sulcus. Evoked extracellular fEPSPs were recorded using an Axoclamp-2B amplifier (Axon Instruments) with an ACSF-filled glass micropipette pulled on a vertical puller (Narishige PC-10, resistance [<5 MU]), inserted in layers II/III at around 500 μm from the stimulation electrode and were analyzed using Axoscope 8.0 software. Baseline responses were obtained every 30 s with the stimulus intensity adjusted to induce 50% of the maximal synaptic response. After 20 min of stable baseline, LTP was evoked by TBS (100 Hz; four sets of stimulations delivered 15 s apart, each one consisting of ten bursts of five pulses at 100 Hz with inter-burst intervals of 150 ms). The fEPSPs were plotted as amplitude. Each point represented the responses every 30 s expressed as the means ± SEM.

### 2.6. Immunohistochemistry

Brain slices were fixed in 4% PFA for 1 h after recording. Slices were treated with 1% Triton X-100 for 20 min, blocked with 3% BSA in PBS for 1 h and incubated overnight, free-floating, with primary antibodies (α-RFP, Rockland Antibodies, Cat#600-401-379, IHC 1:1000; α-TrkB, Santa Cruz, Cat#sc-12g, IHC 1:300; α-βGal, Promega Cat#Z3781, IHC 1:300; α-NeuN, Millipore, Cat#MAB377, IHC 1:1000; α-PSD95, Millipore, Cat#MABN1190 IHC 1:500; α-GFAP Abcam, Cat#ab4674, IHC 1:1000) diluted in blocking buffer. Slices were washed in PBS and incubated for 2 h at room temperature with the secondary antibody diluted in blocking buffer. Slices were eventually counterstained with DAPI and mounted with Aqua-Poly/mount.

### 2.7. Confocal Microscopy

Confocal imaging was performed using a laser-scanning motorized confocal system (Nikon A1) equipped with an Eclipse Ti-E inverted microscope and four laser lines (405, 488, 561 and 638 nm). Z-series images were taken with an inter-stack interval of 0.5 µm using a 60X oil objective. Image processing and 3D rendering were performed using the software NIS Element (Nikon Europe B.V., Amsterdam, The Netherlands).

### 2.8. Structured Illumination Microscopy (SIM)

SIM was performed using an X-Light V2 confocal spinning disk system complete with a Video Confocal super-resolution module (CrestOptics, Rome, Italy) with a lateral resolution of 115 nm and an axial resolution of ~250 nm. The system was equipped with a 60×/1.40 NA Plan Apo Lambda Oil Immersion Objective (Nikon Europe B.V., Amsterdam, The Netherlands), and a Spectra X Lumencor LEDs Light Source with bandpass excitation filters of 460–490 and 535–600 nm (Chroma Technology, Rockingham, Vermont, USA). Image stacks were acquired with a format of 2048 × 2048 pixels, a z-distance of 150 nm and 36 raw SIM images per plane (multiple acquisition mode x–y grid scan). The SIM raw data with 16-bit depth were computationally reconstructed using the Metamorph software package. For 3D image rendering and colocalization analysis, images were processed using NIS-Elements software.

### 2.9. Statistical Analysis

Power analysis was applied to calculate the minimum sample size for LTP recordings (*n* = 10 slices, 6 mice from TrkB^lox/lox^; *n* = 9 slices, 6 mice from control littermates; *n* = 10 slices, 6 mice from TrkB^lox/lox^-Cre; *n* = 11 slices, 6 mice from control littermates). Data were summarized by mean ± SEM. LTP data were analyzed using two-way ANOVA (5–10 min before TBS; 0–5 min post-TBS; 115–120 min post-TBS; and 175–180 min post-TBS) followed by the Bonferroni post-test.

## 3. Results

**Figure 1:**
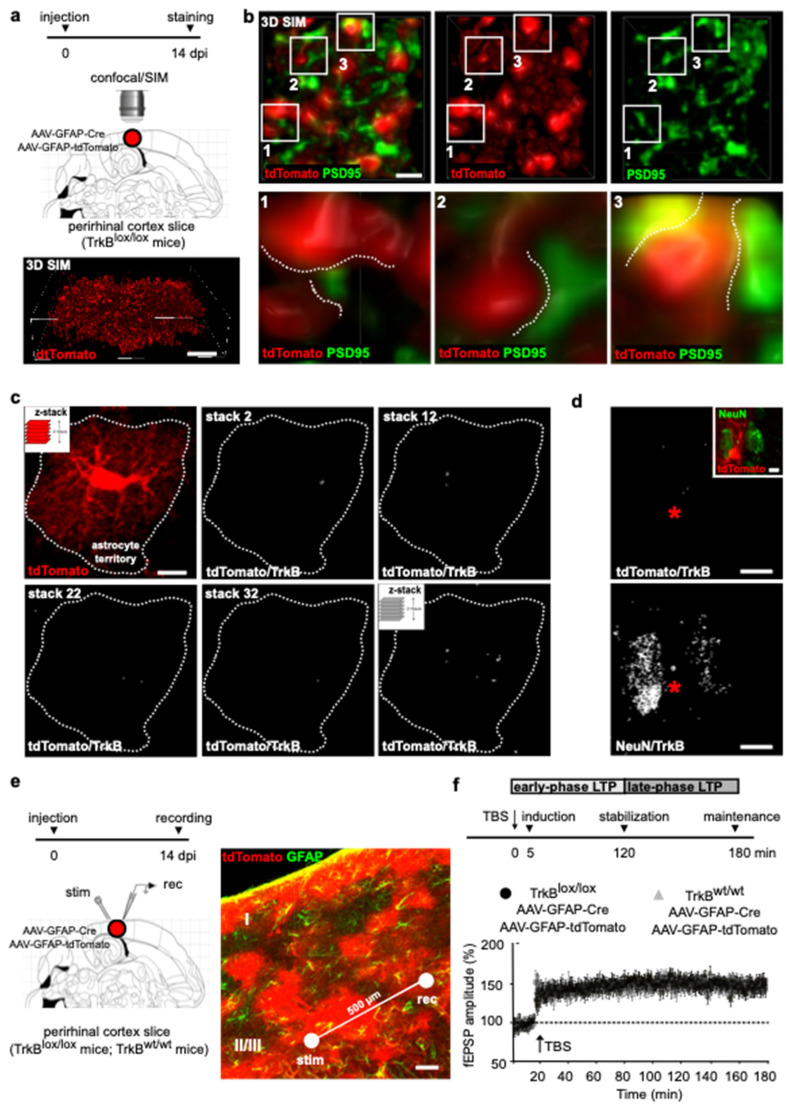
LTP evoked in cortical slices from TrkB^lox/lox^ mice injected with AAV-GFAP-Cre. (**a**) Schematic diagram depicting the experimental paradigm (upper). TrkB^lox/lox^ mice were double-injected with AAV-GFAP-Cre and AAV-GFAP-tdTomato in layers II/III of the perirhinal cortex and slices were prepared for immunostaining 14 dpi. Three-dimensional-SIM reconstruction of an astrocyte labeled with tdTomato (lower). Scale bars: 10 µm. (**b**) Upper panels show astrocyte microdomains labeled by tdTomato near post-synaptic sites labeled by PSD95 (left). Individual tdTomato (middle) and PSD95 (right) signals are shown. Lower panels show five-times magnifications of astrocyte microdomains (white squares) shaped as lamellar sheet (1) and finger-like extension (2 and 3) contacting (dashed lines) pre-synaptic structures labeled by PSD95. Scale bar: 1 µm. (**c**) Representative confocal image of an astrocyte co-transduced with AAV-GFAP-Cre and AAV-GFAP-tdTomato (left); dashed line depicts the astrocyte territory. Stack-by-stack reconstruction of tdTomato/TrkB colocalization signals from the same astrocyte (right); selected stacks 2, 12, 22 and 32, and final z-stack reconstruction of tdTomato/TrkB are shown. Scale bar: 10 µm. (**d**) Representative confocal image of one astrocyte (asterisk) surrounded by 2 neurons labeled by tdTomato and NeuN, respectively (inset). The z-stack reconstruction of tdTomato/TrkB and NeuN/TrkB colocalization signals of the same image. Scale bar: 10 µm. (**e**) Schematic diagram depicting the experimental paradigm (left). TrkB^lox/lox^ mice were double injected with AAV-GFAP-Cre and AAV-GFAP-tdTomato in layers II/III of the perirhinal cortex and slices were prepared for field recording 14 dpi. Confocal image depicts the perirhinal cortex labeled with tdTomato and GFAP (right). A concentric bipolar electrode was positioned within the injected area in layers II/III on the temporal side of the rhinal sulcus (stim). Evoked extracellular fEPSPs were recorded using ACSF-filled glass micropipette (rec) placed at around 500 µm (white line) from the stimulation electrode. (**f**) Schematic diagram depicting the experimental paradigm (upper). LTP induction, consolidation and maintenance were analyzed 5 min, 120 min and 180 min after TBS, respectively. Temporally defined early phase and late-phase LTP are shown. Lower panel shows fEPSPs recording plotted as amplitude. After 20 min of stable baseline, LTP was evoked by TBS. Data are mean ± SEM. (*n* = 10 slices, 6 mice from TrkB^lox/lox^; *n* = 9 slices, 6 mice control littermates). Two-way ANOVA (F time x genotype (3.51) = 2.088, *p* = 0.1133; F time (2.055, 34.94) = 44.42, *p* < 0.0001; F genotype (1, 17) = 0.02019, *p* = 0.8887) on average data between 5 and 10 min before TBS had mean (control littermates, TrkB^lox/lox^) = 99.30, 102.5; *p* > 0.9999 Bonferroni post hoc test; 0–5 min post-TBS mean (control littermates, TrkB^lox/lox^) = 146.40, 131.0; *p* = 0.8129 Bonferroni post hoc test; 115–120 min post-TBS mean (control littermates, TrkB^lox/lox^) = 148.30, 151.4; *p* > 0.9999 Bonferroni post hoc test; and 175–180 min post-TBS mean (control littermates, TrkB^lox/lox^) = 144.2, 150.2; *p* > 0.9999 Bonferroni post hoc test.

To investigate the contribution of astrocyte TrkB signaling to cortical LTP, we targeted the *TrkB* gene to protoplasmic astrocytes expressing the prototypical astrocyte marker glial fibrillary acidic protein (GFAP). Homozygous TrkB^lox/lox^ mice, in which the second exon of the TrkB tyrosine kinase region had been flanked by two loxP sites [12], were co-injected in layers II/III of the perirhinal cortex with two adenoviral constructs, transducing Cre recombinase and the reporter tdTomato under the control of the GFAP promoter (AAV-GFAP-Cre; AAV-GFAP-tdTomato [13]). Slices were prepared 14 days post-injection (dpi) and cortical sections were stained for tdTomato (Figure 1a). Astrocytes showed highly branched arborization and fine membrane extensions at the cell peripheries (Figure 1a), a typical morphology at this cortical layer [14]. Fine arborization delimiting the astrocytes’ territory was then resolved in nanometer-scale resolution (lateral resolution 115 nm; axial resolution 250 nm) using structured illumination microscopy (SIM) (Figure 1a,b). We observed that the membrane ramifications were mostly shaped as finger-like extensions and flat lamellar sheaths, which are recognized to be astrocytic structures contacting synapses [5,15,16], or the so-called microdomains [17]. Accordingly, astrocyte microdomains were localized in the proximity of synaptic structures, expressing the post-synaptic marker PSD95 (Figure 1b). Overall, our data indicate that the *TrkB* gene is deleted from astrocytes, shaping membranous elaborations that view both dimensions and identities of peri-synaptic structures.

To evaluate whether astrocytes transducing the viruses are also depleted in TrkB protein, we stained cortical sections using an αTrkB antibody, which targets the intracellular domain of the full-length TrkB, leaving undetected t-TrkB, the truncated isoform of the receptor (Figure 1c). The expression of the receptor was analyzed using confocal microscopy in which TrkB immunoreactivity was overlayed to that of tdTomato in individual astrocytes. tdTomato is a cytosolic protein whose fluorescence defines the astrocyte in its entire cytoplasmic extension. This is a feature that is ideal to achieve detection of TrkB in the whole territorial volume of astrocytes. Spatial overlap of TrkB and tdTomato signals was analyzed in a series of confocal stacks by using co-localization analysis of the two signals and TrkB/tdTomato colocalization reconstructed in z-stacks. We used Mander’s overlap, which measures co-occurrence of the two signals in the astrocytic territory [11]. We adopted co-occurrence as it accounts for the abundance of TrkB that overlaps with tdTomato and it ameliorates the effects of unwanted signals, such as auto-fluorescence or other near-threshold signals. Finally, it can offer an intuitive accounting of the concentrated weighted overlap between TrkB and tdTomato, and it shows great ability to assess to what extent TrkB can be found in the astrocytic territory in an intensity-weighted manner. We showed that the overall amount of TrkB in the astrocytic territory is minute, if not absent, as only background levels of TrkB signal could be detected in tdTomato+ astrocytes at the injected area. On the contrary, higher levels of TrkB appeared in nearby neurons, as revealed by TrkB colocalization with the neuronal marker NeuN (Figure 1d). Thus, we provided clear demonstration that astrocytes co-transduced with AAV-GFAP-Cre and AAV-GFAP-tdTomato are devoid of TrkB protein.

Parallel slices were used for field recording, delivering θ-burst stimulation (TBS) and evoking LTP. Electrophysiological recordings were carried out in layers II/III of the perirhinal cortex, where tdTomato fluorescence was particularly intense (Figure 1e). Stimulation of horizontal fibers at 0.033 Hz evoked a field potential, the amplitude of which did not change during recording (Figure 1f). LTP was induced applying four trains of high-frequency stimulation (100 Hz) separated by an interval of 15 s (Figure 1f). This bursting activity, which is repeated at a frequency of the θ rhythm, represents an electrical activity pattern that mimics the physiological neural activity during learning [18]. LTP induction, consolidation and maintenance were analyzed 5 min, 120 min and 180 min after the stimulation, respectively, which also temporally define the early phase and late phase of synaptic potentiation (Figure 1f). In slices from TrkB^lox/lox^ mice co-injected with AAV-GFAP-Cre and AAV-GFAP-tdTomato, TBS induced an increase in fEPSP amplitude that remained potentiated above baseline for the 3 h duration of the recording. Similar results were obtained in control littermates. Post hoc analysis confirmed that the injection in our slices was layer- and cell-specific, exhibiting that 92 ± 6% of GFAP+ cells expressed tdTomato. Our quantification was necessarily confined to the recording area covering a 500 µm distance between the electrodes that are placed inside the area of injection (Figure 1e). Thus, targeted TrkB deletion, and therefore BDNF-induced TrkB signaling in cortical layer II/III astrocytes, had no effect on LTP induction, stabilization and maintenance.

**Figure 2:**
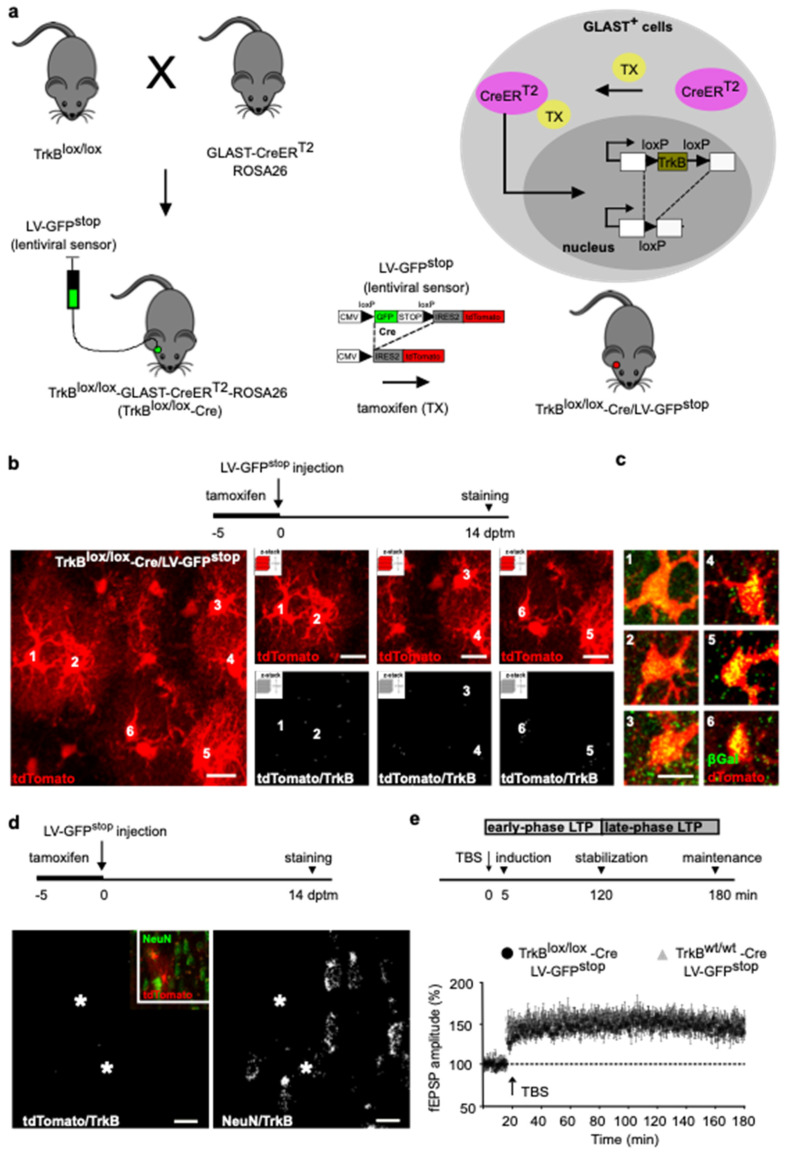
LTP evoked in cortical slices from TrkB^lox/lox^-Cre mice. (**a**) Schematic diagram depicting the experimental paradigm. TrkB^lox/lox^-Cre mice were generated by breeding TrkB^lox/lox^ mice, expressing flanking exons 2 of the TrkB catalytic domain with loxP sites, with GLAST-CreER^T2^ mice, expressing the inducible version of the Cre recombinase (CreER^T2^) under the control of the GLAST promoter, crossed with ROSA26 mice. Tamoxifen treatment in TrkB^lox/lox^-Cre mice causes the CreER^T2^ fusion protein to translocate into the nucleus of GLAST-expressing cells, where it recombines paired loxP sites, allowing for *TrkB* gene deletion and bGal expression. To assess recombination at the recording area, TrkB^lox/lox^-Cre mice were injected with LV-GFP^stop^. A loxP-GFP-STOP-loxP cassette allows for GFP expression while preventing that of tdTomato. The GFP-STOP cassette was deleted by Cre recombinase, resulting in GFP loss and activation of the tdTomato reporter. (**b**) Schematic diagram depicting the experimental paradigm (upper). TrkB^lox/lox^-Cre mice were treated with tamoxifen (−5 to 0 dptm), injected with LV-GFP^stop^ (0 dptm), and the slices were prepared for staining after 14 days (14 dptm). Lower panels show a representative confocal image of astrocytes labeled by tdTomato (left). The z-stack reconstruction of tdTomato and tdTomato/TrkB colocalization signals of astrocytes 1 to 6 from the same image (right). Scale bar: 10 µm. (**c**) Astrocytes 1 to 6 as in b, stained for tdTomato and bGal. Scale bar: 10 µm. (**d**) Schematic diagram depicting the experimental paradigm as in **b** (upper). Representative confocal image of astrocytes (asterisks) and nearby neurons labeled by tdTomato and NeuN, respectively (inset). The z-stack reconstruction of tdTomato/TrkB and NeuN/TrkB colocalization signals of the same image. Scale bar: 10 µm. (**e**) Schematic diagram depicting the experimental paradigm (upper). LTP induction, consolidation and maintenance were analyzed 5 min, 120 min and 180 min after TBS, respectively. Temporally defined early phase and late-phase LTP are shown. Lower panel shows fEPSPs recording plotted as amplitude. After 20 min of stable baseline, LTP was evoked by TBS. Data are mean ± SEM. (*n* = 10 slices, 6 mice TrkB^lox/lox^-Cre mice; *n* = 11 slices, 6 mice control littermates; two-way ANOVA. (F time × genotype (3.57) = 0.6397, *p* = 0.5926; F time (2.338, 44.41) = 64.79, *p* < 0.0001; F genotype (1, 19) = 1.632, *p* = 0.2168) on average data of 5-10 min before-TBS mean (control littermates, TrkB^lox/lox^-Cre) = 101.5; 101.1; *p* > 0.9999 Bonferroni post hoc test; 0–5 min post-TBS mean (control littermates, TrkB^lox/lox^-Cre) = 146.6, 135.8; *p* = 0.9901 Bonferroni post hoc test; 115–120 min post-TBS mean (control littermates, TrkB^lox/lox^-Cre) = 154.8, 149.5; *p* > 0.9999 Bonferroni post hoc test; and 175–180 min post-TBS mean (control littermates, TrkB^lox/lox^-Cre) = 151.4, 142.5; *p* = 0.7101 Bonferroni post hoc test).

In adult mice, astrocytes are present in all cortical areas and neuronal layers [19,20]. Neurons in each layer of the cortex have distinct properties and connections, and astrocyte diversity is shaped by local interactions with neurons [21], thereby astrocytes might be expected to support and modify neuronal circuitry in a cellular and layer-specific manner [20]. Given that GFAP-positive astrocytes are just a subset of the entire astrocyte population, we extended *TrkB* gene deletion to include astrocytes expressing the glial-specific glutamate transporter (GLAST), a marker for most astrocytes across brain regions [20]. We used a mouse line expressing the inducible form of Cre (CreER^T2^) in the GLAST locus [22], crossed with ROSA26 mice for βGalactosidase (βGal) expression. The resulting mice were further crossed with homozygous TrkB^lox/lox^ mice. Tamoxifen treatment in this mouse line (from now on TrkB^lox/lox^-Cre) caused the CreER^T2^ fusion protein to translocate into the nucleus of GLAST-expressing cells, where it recombined paired loxP sites and removed the catalytically active *TrkB* gene (Figure 2a) [23,24]. To confirm recombination at the recording site, we injected LV-GFP^stop^ lentivirus construct [10] in layers II/III of the perirhinal cortices of TrkB^lox/lox^-Cre mice. In this sensor construct, a loxP-GFP-STOP-loxP cassette allows for GFP expression, while preventing tdTomato expression (Figure 2a). In the presence of Cre recombinase, the GFP-STOP cassette is removed, resulting in GFP loss and expression of the tdTomato reporter. TrkB^lox/lox^-Cre mice were treated with tamoxifen for 5 days and injected with LV-GFP^stop^ on the last day of tamoxifen treatment (dptm). Cortical slices were prepared 14 dptm; at this time, only tdTomato+ cells were observed (Figure 2b) and 98 ± 3% of them expressed β-Gal (Figure 2c). Moreover, all GLAST+ cells transduced with the lentiviral sensor showed background levels of tdTomato/TrkB colocalization signals (Figure 2b), which contrasted with the higher levels of NeuN/TrkB colocalization signals observed in nearby neurons (Figure 2d). Our data indicate that TrkB^lox/lox^-Cre mice allowed for astrocyte-selective targeting of the receptor.

Slices from TrkB^lox/lox^-Cre mice were used for field recording, placing the electrodes inside the region expressing the lentiviral sensor and assessing LTP. In agreement with the above observations (Figure 1f), we demonstrated that TBS elicited an LTP that lasted for the whole 180 min recording (Figure 2e). Similar results were obtained in control littermates (Figure 2e). Thus, TrkB deletion had no effect on LTP at any stage of synaptic potentiation, indicating that this receptor-signaling in astrocytes is not essential for synaptic strengthening.

## 4. Discussion

Astrocyte functions are shaped by local interactions with neurons [2,3,4,5,6]. This raises the question of how morphological, molecular and functional properties of astrocytes are influenced by changes in the neuronal environment. BDNF is supplied by neurons and represents one of the major players in the reciprocal neuron–astrocyte communication. However, several discrepancies have emerged about the expression of BDNF receptors by astrocytes and their correlated effects, preventing a clear-cut consensus about the role of BDNF in astrocyte functions.

In the present study, we hypothesized that BDNF would exert a permissive action on cortical LTP, involving astrocyte TrkB activation. To address this possibility, fEPSP recordings were attained in cortical layers II/III of the perirhinal cortex from two different experimental settings. First, we used TrkB^lox/lox^ mice in which TrkB deletion was reached by transducing AAV-GFAP-Cre at the site of recording; this strategy targets most GFAP astrocytes with layer specificity [14]. Second, we used conditional TrkB^lox/lox^-Cre mouse line, which is known to target most GLAST-expressing cells with high specificity. GLAST is a protein expressed in most cortical astrocytes, its processes extending near synapses, thereby affecting cells that are part of the tripartite synapse. In both experimental paradigms, we observed no effect of TrkB deletion on synaptic strengthening, excluding astrocyte TrkB signaling from cortical LTP. Thus, it seems unlikely that there would be a single mechanism that regulates BDNF activity on astrocytes for LTP, warranting additional consideration of the mechanisms of BDNF signaling in neuron–astrocyte communication.

Different BDNF receptors are involved in transducing astrocyte functions in LTP. We have previously demonstrated that astrocytes recruit p75^NTR^ for LTP maintenance. We showed that BDNF is secreted from neurons in its pro-form (proBDNF) and is internalized by astrocytes via p75^NTR^-mediated endocytosis [10,25,26]. The endocytic proBDNF is then processed by astrocytes, which converts the inactive precursor in the prodomain and mature BDNF, and releases these proteolytic products for synaptic re-use. Upon proBDNF recycling, both prodomain and mature BDNF act to reinforce TrkB signaling at post-synaptic sites, activating adaptive molecular mechanisms that promote LTP maintenance and memory consolidation [10,11]. Thus, a time-sensitive increase in BDNF availability is required for LTP maintenance, and proBDNF recycling by glial cells can compensate for this physiological requirement. This process involves the conversion of proBDNF from inactive to a multi-functional state, indicating the presence of local information storage in astrocytes for supporting memory circuits.

In contrast to proBDNF recycling, other studies suggest that solely the mature BDNF is endocytosed by astrocytes via full-length TrkB [27] or truncated t-TrkB receptors [28]. In the first case, the catalytically active TrkB orchestrates BDNF recycling, through which BDNF is initially internalized and then re-released [27]. In the second case, the truncated t-TrkB receptor functions to subtract BDNF from synaptic signaling, terminating its action and re-directing the endocytic neurotrophin to a degradation pathway [28]. These conflicting mechanisms could be due to differences in experimental conditions such as: (i) cultured astrocytes [27] vs. acute slices [10,25]; (ii) cortical [10,25] vs. hippocampal astrocytes [27,28]; or (iii) different stimulation paradigms, implying that BDNF signaling on astrocytes is dependent on the intensity of neuronal stimulation [29]. These important aspects must be addressed to clarify current inconsistencies; however, despite the present uncertainty, our study clearly excludes the participation of full-length TrkB in mediating astrocyte signaling for perirhinal cortex LTP.

The control of BDNF availability, in relation to neuron–astrocyte communication, might also count on BDNFs skill to induce BDNF release via TrkB activation [30,31,32]. This self-amplifying autocrine action of BDNF has been suggested to increase the ready-releasable pool of BDNF, and to involve astrocytes in the participation of synaptic strengthening [9]. While BDNF-induced BDNF release was demonstrated to act as a positive, reinforcing mechanism in diverse modes of neuronal functions, such as neuronal precursor migration in the cerebellum [33] or axonal specification and growth in developing neurons [34], local, self-amplifying BDNF secretion from astrocytes does not seem to play a critical role in astrocyte-signaling for cortical LTP.

Astrocytes can sense extracellular BDNF for local translation [35]. In neurons, activity-dependent translation of mRNA into proteins is essential for long-term changes in synaptic strength and efficacy, suggesting that BDNF-mediated translational responses in peri-synaptic astrocytes might contribute to LTP maintenance. The change in transcripts on astrocyte ribosomes is highlighted by a rapid increase in transcripts related to cytoskeletal dynamics, motor activity, ion transport and cell communication. This indicates a set of dynamic responses, some of which may be secondary to activation of TrkB signaling. Our findings indicate that this process is extraneous to the regulation of cortical LTP. Alternative signaling pathways, inducing protein synthesis in astrocytes, may integrate synaptic strengthening, such as ion concentration changes (high K+) that are known to occur in the synaptic cleft in response to neuronal activity [35].

In conclusion, the role of BDNF in synaptic strengthening is a hotly debated issue, and some contradictory observations exist, casting doubt on how astrocytes sense or release BDNF to modulate synaptic potentiation. The debates mainly revolve around the different expression of BDNF receptors through which astrocytes provide specific signaling for LTP. Our previous [10,11,25] and present work indicate that astrocytes play an important role in the regulation of synaptic strengthening by BDNF, but this regulation does not involve astrocyte TrkB signaling. Thus, our study sheds new light on BDNF function in astrocyte-mediated regulation of cortical LTP, excluding TrkB signaling as a potential mechanism.

## Data Availability

The data presented in this study are available from the corresponding authors upon request.
